# Toward an intragroup approach to alleviate ageism in the second half of life

**DOI:** 10.1093/geront/gnaf169

**Published:** 2025-07-28

**Authors:** Liat Ayalon

**Affiliations:** Louis and Gabi Weisfeld School of Social Work, Bar Ilan University, Ramat Gan, Israel

**Keywords:** Age discrimination, Intergroup conflict, Social identity theory, Ageism, Older adults

## Abstract

Ageism, defined as stereotypes, prejudices, and discrimination based on age, is highly prevalent and has negative health and mental health impacts. Although interventions to reduce ageism directed by younger agents of ageism toward older persons exist, there is scarcity of knowledge concerning interventions to alleviate ageism directed by older persons toward other older persons or toward themselves. The proposed intragroup approach to ageism challenges the traditional view of older persons solely as victims of ageism, highlighting their role as both agents or targeters and targets of ageism—thus, exhibiting self-directed and other-directed ageism, pointed toward themselves and toward other older persons, respectively. The intragroup approach emphasizes the complexity of ageism in the second half of life, when bias and conflict occur within the group of older persons rather than between groups, thus requiring a nuanced understanding of one’s subjective social group identification. The proposed theoretical framework identifies needed steps to transition toward personalized interventions of ageism, which affect change by matching interventions to the characteristics of the individual and the context in which ageism operates. This includes attention to the relationships between self-and other-directed ageism, as determined by subjective social group identification, the multidimensional nature of ageism, as composed of stereotypes, prejudices, and discrimination including an implicit component, and the possible impact of contextual factors, related to the prominence of ageism in society.

## Background

Ageism is defined as the way we think, feel, and act toward people because of their age. It is manifested in stereotypes, prejudices, and discrimination (Ayalon & Tesch-Römer, 2018). Although ageism can be directed toward people of all ages, most research evidence is focused on older persons as the targets of ageism (Ayalon & Tesch-Römer, 2017). This line of research has stressed the pervasiveness of ageism and its detrimental effects on the health and well-being of older persons (Chang et al., 2020). In the theoretical approach presented in this article, I claim that the focus on ageism directed toward older persons by younger agents of ageism has contributed to the view of older persons as victims of ageism and disregarded older persons as possible targeters or agents of ageism. Ageism is not only characterized by younger persons holding ageist attitudes, behaviors, and feelings toward those who are older than they are, but also by older persons holding ageist attitudes, behaviors, or feelings against themselves (self-directed ageism) or against their same-age peers (other-directed ageism) (Ayalon & Tesch-Römer, 2017; Levy, 2009). Older persons as the agents of ageism represent a unique phenomenon (Nelson, 2011). To fully capture such a phenomenon, there is a need to supplement an intergroup approach (one group against another) (Stephan & Stephan, 2017) with an intragroup approach (relations of targets and targeters occur within the same group). To do so, the intragroup approach draws on two major theories in the field of ageism: social identity theory and stereotype embodiment theory.

### Current theoretical approaches to explain ageism

#### Social identity theory

According to social identity theory, people think of themselves not only as individuals, but also as group members, using social categorization, social comparison, and social identification to define their social group membership which in return shapes intergroup relations (Tajfel, 1974). To protect their self-worth, people may mobilize to a different social group which is perceived as having a more positive value. They may also attempt to represent their own social group in a more positive light or engage in conflict to change the status quo between groups (Tajfel & Turner, 2004).

Consistent with the predictions of social identity theory, older persons may disassociate from the older persons’ ingroup by holding a younger subjective age (e.g., how old one feels) than their chronological age (Okun & Ayalon, 2023b). Indeed, there is plenty of research to show that older persons tend to hold a younger subjective age, viewing themselves as younger than they actually are (Kotter-Grühn et al., 2015; Rubin & Berntsen, 2006). In fact, research has found a gap between objective chronological age and subjective age in older persons, as older persons consistently tend to feel younger than their chronological age by about 13%–18% (Pinquart & Wahl, 2021). Older persons who hold a younger subjective age are healthier, enjoy better well-being, and even have a longer life expectancy (Deeg et al., 2021; Stephan et al., 2021; Veenstra et al., 2021). Hence, holding a younger subjective age is protective of older persons.

Nonetheless, in the absence of subjective social group identification as older persons, older persons are less likely to become their own self-advocates. Such an approach was documented in the case of the climate change movement, when older persons’ organizations advocate for younger persons, largely neglecting to acknowledge their own susceptibility in the face of the changing climate (Aharoni Lir & Ayalon, 2024). Likewise, the protests that took place in Israel in response to the judicial overhaul were largely led by older persons, who emphasized their motivation to secure the future of their children and grandchildren, while neglecting the impact of the judicial overhaul on their own lives (Ayalon & Okun, 2024a, 2024b).

#### Stereotype embodiment theory

The stereotype embodiment theory has stressed the negative impact of societal stereotypes on older persons via their internalization and self-relevance (Levy, 2009). Ageism is different from sexism or racism. Not all of us are women or belong to a minority group, but we all have an age. The problem with ageism is that it is so prevalent and pervasive that we hardly question it and often fail to notice its presence (Levy & Banaji, 2002). Because ageism penetrates our mindset at an early age, when we grow older, negative stereotypes of older persons become self-relevant and older persons direct ageist stereotypes toward themselves (Levy, 2001; Minichiello et al., 2000).

#### Intergroup approach

Research has largely relied on intergroup processes to explain stereotypes, prejudices, and discrimination in the contexts of sexism or racism (Halperin et al., 2023). This theory also has been used to explain ageism directed by younger age groups toward older persons (Lev et al., 2018). Simply put, according to the intergroup approach, we are biased against those who are not part of our ingroup.

**Figure 1.**
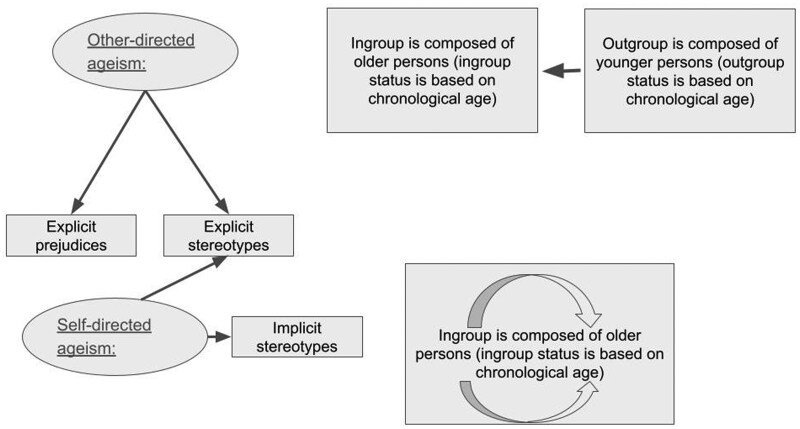
Current interventions to address other-and self-directed ageism from an intergroup perspective.

Figure 1 illustrates current understanding of ageism and interventions to address ageism from an intergroup approach. The intergroup approach views older persons as the targets of ageism and younger persons as agents, with the exception of self-directed ageism by older persons toward themselves, as predicted by the stereotype embodiment theory (Levy, 2009).

Current interventions to reduce self-directed ageism have largely relied on the stereotype embodiment theory, targeting other-directed ageism implicitly and showing an improvement in self-directed ageism, following the internalization of positive views on aging (Levy et al., 2014). Other interventions focused on physical exercises, mindfulness, acceptance, and/or educational information to alleviate self-directed ageist stereotypes (Zhu et al., 2025). Interventions to reduce other-directed ageism among older persons in the second half of their lives have relied mainly on education (Doncel-García et al., 2023). From research on younger persons as the agents of ageism, we know that interventions are effective in reducing ageist prejudices and stereotypes (Apriceno & Levy, 2023; Burnes et al., 2019).

### The need for an intragroup approach to ageism

In this article, I argue for the need for an intragroup approach to understand ageism in the second half of life (defined as age 60 and over by the World Health Organization [WHO]). There is plenty of evidence to show that older persons hold ageist stereotypes, prejudices, and discrimination not only toward themselves, but also toward other older persons, who are their peers (Ayalon, 2015; Ayalon & Tesch-Römer, 2017; Dobbs et al., 2008; Giasson et al., 2017; Lagacé & Firzly, 2017). Research has shown that ageism directed toward older persons by older persons is as prevalent or more compared with ageism directed by younger persons toward older persons (Cherry et al., 2016; Cohn-Schwartz & Ayalon, 2021). Likewise, self-directed ageism is highly prevalent in multiple contexts and cultural groups (Hausknecht et al., 2020; Tully-Wilson et al., 2021).

The negative impact of ageism directed by agents in the second half of their life is well-established. In fact, self-directed ageism has a more negative impact on health and well-being outcomes than any other type of ageism (Chang et al., 2020; Tully-Wilson et al., 2021). One proposed mechanism for the negative effects of self-directed ageism on one’s health and well-being is via a reduction in self-efficacy and health behaviors, resulting in a self-fulfilling prophecy (Levy & Myers, 2004; Wurm et al., 2013).

Likewise, there is plenty of research to show that ageism directed by older persons toward other older persons is detrimental to older persons’ social participation and sense of loneliness (Ayalon, 2018; Schwartz et al., 2021). For instance, research has shown that negative self-directed ageism mediates the relationship between exposure to ageism and loneliness in older persons (Terán-Mendoza & Cancino, 2025). A different study identified anxiety about aging as a mediator, which accounts for the relationship between care settings and sense of loneliness (Ayalon, 2018). This association with anxiety about aging is relevant not only for older persons’ sense of loneliness, but also for middle-aged adults, who are more likely to report loneliness, cooccurring with anxiety about aging (Bergman & Segel-Karpas, 2021).

### Current gaps in research and theory

The intergroup approach has been a harbinger to many interventions advocating for increased contact and collaboration between people of different groups to reduce stereotypes, prejudices, and discrimination (Pettigrew et al., 2011). Supposedly, by working together toward a common goal, people begin to view themselves as more similar to those who were previously seen as outgroup members. Under such circumstances, the distinction between us versus them becomes blurred, and the bias which characterizes previous interactions between people of different groups is reduced.

But what happens when bias or marginalization occurs within a single group, as in the case of older persons? How can we explain this bias if it is no longer reflective of a clear-cut distinction between one group versus another? Under such circumstances, there is a need to add an intragroup approach. An intragroup approach views older persons as belonging to the exact same group which they are biased against. This may result in self-directed and/or other-directed ageism, occurring within a single group of people arbitrarily defined as “older persons” by society, based on their chronological age. Below, I discuss the role of social group identity, the multidimensional nature of ageism, and the context in which ageism takes place as areas requiring further research to improve the understanding of ageism, especially in the second half of life.

#### Social group identity

Holding a younger age group identity than one’s chronological age can be beneficial for older persons. However, this may reflect ageism toward other older persons (e.g., using a comparison which devalues the perceived “other”). For instance, research has found that older persons often describe themselves as younger than their chronological age, alluding to their positive aging process, but at the same time, also acknowledge their negative beliefs about older persons in general. Statements such as, “I am 70 years old, but I feel like I am not even 50,” “I am young at heart,” or “even though I am already 80, I am still active in good spirit” are supposed to reflect a positive view of one’s aging experiences and indeed, they reflect a younger subjective age. However, they also reflect inherent assumptions about older age as time of decline and limitations, which may not be expressed directly but can be inferred between the lines (Okun & Ayalon, 2023b).

The liminal nature of older persons’ social group identification is notable. For instance, older persons may feel younger than their chronological age, yet, self-identify with their generational group (which remains constant throughout the life course) (Weiss & Lang, 2012). This represents a process through which older persons make sense of their own social group identity using means of distancing, adapting, and redefining (Schuurman et al., 2024). It stresses the complexity of older age group identification and the need for an intragroup approach, which views older persons as belonging to the in-group based on their older chronological age, while acknowledging the fluidity inherent in their subjective social group identification.

Ageism is different from other forms of marginalization. In the case of other forms of marginalization, identifying with one’s group has a positive impact even when the group is experiencing discrimination (Jetten et al., 2017). For instance, using an experimental design, researchers found that being informed about group disadvantage can ameliorate, rather than intensify one’s own experiences of personal rejection (Stroebe et al., 2009). This may not be the case for ageism, as older persons attempt to disassociate themselves from the older persons’ age group category (Okun & Ayalon, 2023b). The difference found between ageism and other marginalized social groups could be attributed to the relative fluidity of having an older age identity. Compared with other marginalized identities, our chronological age changes constantly, allowing for greater permeability between the ingroup of older persons and the outgroup of younger persons.

#### The multidimensional nature of ageism

Another major shortcoming of past research concerns the limited attention to the multidimensional nature of ageism. The most comprehensive definition of ageism concerns all three dimensions: stereotypes, prejudices, and discrimination, acknowledging its explicit and implicit nature (Iversen et al., 2009). However, to the most part, interventions in the second half of life targeted self-directed ageist attitudes with a minority of studies also targeting other-directed ageist attitudes (Knight et al., 2022). We know from research conducted with younger persons as agents of ageism that our ability to impact all three dimensions of ageism is limited (Apriceno & Levy, 2023; Burnes et al., 2019). This is also true for research attempting to address intergroup conflict in other domains which resulted in limited ability to influence behaviors (Brauer, 2024).

Research on interventions to reduce other intergroup conflicts proposes a possible connection between the mechanism of action of the intervention and its main outcome (e.g., cognitive interventions impact the cognitive domain) (Galinsky & Moskowitz, 2000; Turner et al., 2007). Such an exploration was not conducted in the context of ageism, given the almost exclusive focus on ageist attitudes in past research. Likewise, a clear distinction between implicit versus explicit interventions and their impact on the individual was not adequately performed in the field of ageism (Ayalon et al., 2023; Tseng et al., 2025).

In addition to these important considerations concerning the mechanism of action and its impact on outcomes, it is also important to consider the person or the agent of ageism to ensure the best fit between interventions and outcomes. Although personalized medicine, which fosters the matching of patient characteristics to treatment, is becoming the norm (Goetz & Schork, 2018), this is not the case in the field of ageism. We still lack knowledge as to which intervention works for whom and why.

The compensation/capitalization theory suggests that interventions can be matched to meet people’s weaknesses or assets, respectively, to generate a stronger effect (Wingate et al., 2005). Hence, it is possible that being particularly high or low on ageist stereotypes may require an intervention that relies on a cognitive mechanism, whereas being particularly high or low on ageist prejudices may require an intervention that relies on an emotional mechanism. Research will benefit from developing such a nuanced approach to the study of ageism.

#### The context of ageism

Context is another unexplored factor which is expected to determine the type of intervention needed and its possible effectiveness. We know that ageism varies in different contexts, though it is still not entirely clear what context characteristics are most important in determining the occurrence of ageism (North & Fiske, 2015; Officer et al., 2020), let alone the effectiveness of potential interventions to alleviate ageism in different contexts. This is because, in the field of ageism, most interventions were developed in the West, primarily in North America (Apriceno & Levy, 2023; Burnes et al., 2019). From intervention research that addresses other “isms,” we know that the degree of conflict in society, the degree of group integration versus segregation, and social norms are important contextual features (Čehajić-Clancy & Halperin, 2024).

The prevalence of ageism varies over the lifespan, with some countries such as the United Kingdom, showing particularly high levels of ageism in younger persons, versus the Czech Republic showing higher levels of ageism in older persons, or Israel, which is characterized by a U-shaped distribution (Bratt et al., 2018). It is possible that the distribution of ageism across the lifespan is a factor that requires consideration as this can be indicative of the status of younger versus older persons in society. However, further research is needed to fully understand the contextual effects on interventions.

### An intragroup approach to ageism in the second half of life

The intragroup approach to ageism in the second half of life suggests that although older persons are arbitrarily classified by society as belonging to a single group of older persons, defined by reaching a certain chronological age, their social group identification, operationalized here as subjective age and the degree of permeability of relations with other age groups determine their approach to other older persons and to themselves as representing an ingroup of older persons or a perceived (subjective) outgroup. The intragroup approach further stresses the importance of acknowledging all three dimensions of ageism (e.g., stereotypes, prejudices, and discrimination), including its implicit (unconscious) component and the context in which ageism takes place to better understand how change in ageism occurs and how to effectively alleviate ageism in the second half of life. Table 1 outlines the main terms used to conceptualize the intragroup approach to ageism in the second half of life.

**Table 1.** Key terms used to conceptualize the intragroup approach to ageism in the second half of life.

Concept	Definition
Ageism	Stereotypes, prejudices, and discrimination toward people because of their age. Can be either self- or other-directed
Self-directed ageism	Stereotypes, prejudices, and discrimination toward oneself because of one’s age
Other-directed ageism	Stereotypes, prejudices, and discrimination toward others because of their age
Ageist stereotypes	Oversimplified or biased thinking toward people because of their age
Ageist prejudices	Oversimplified or biased feelings toward people because of their age
Ageist discrimination	Oversimplified or biased behaviors toward people because of their age
Implicit ageism	Ageism experienced unconsciously with limited awareness
Subjective age	The way one feels about his or her age, rather than one’s chronological age
Intergroup relations	Formed between two distinct groups of people
Intragroup relations	Formed within a single group of people
Social group identity	The sense of belonging to a certain social group

In the case of older persons, ageism is not only a product of intergroup relations, directed by younger age groups toward older persons, but also a product of intragroup relations, with the intragroup being artificially constructed by society based on reaching a certain chronological age. Ageism in the second half of life is unique: It is targeted by older persons toward themselves and toward other older persons, within their chronological age group, rather than between two different groups. Hence, it cannot be fully captured by the paradigm of intergroup relations (Stephan & Stephan, 2017; Tajfel & Turner, 2004), which is used to explain bias and marginalization as representing conflict and tension between two distinguished groups. The intragroup approach is needed given the fact that regardless of older persons’ subjective social group identification, they are identified by society as old once they reach a certain chronological age (Higgs & Gilleard, 2021).

An intragroup approach, which considers the relationships between self- and other-directed ageism, the multidimensional nature of ageism, including its implicit component, and the context in which ageism takes place is highly needed. Intervention research should consider subjective social group identification, operationalized as subjective age and degree of permeability across age groups, as well as the preintervention level of ageism on its varied dimensions and implicit component to alleviate ageism. Although context matters, how it matters in the field of ageism interventions remains underexplored. We must identify what context characteristics are of utmost relevance for determining interventions’ effectiveness and how to go beyond the particularities of the context for large-scale implementation of selected interventions globally.

Although not yet established by research, I suggest that one’s subjective age and the permeability of relations across age groups are important components which determine whether ageism directed toward older persons by older persons represents within-group processes or perceived between-group processes. When older persons hold a younger subjective age and disassociate themselves both mentally and possibly physically from other older persons by establishing stronger relationships with younger age groups, it is likely that their ageism toward other older persons is more pronounced than their self-directed ageism. This represents a unique case of subjective or felt intergroup relations, which occur in the face of objective intragroup relations. In contrast, when older persons view themselves as older persons, thus hold a subjective age, which is consistent with their chronological age or older than their chronological age, they may be more likely to experience self-directed ageism, rather than other-directed ageism, as they see themselves as belonging within the same group of older persons. An intragroup approach views older persons as agents and targets of self-and other-directed ageism within the single group of older persons.

Drawing from social identity approaches (Tajfel, 1974; Tajfel & Turner, 2004), the intragroup approach to ageism considers the subjective view of the agents as belonging (or not) to the older persons’ age group as highly important. It is hypothesized that when the agents view themselves as belonging to the ingroup of older persons, they are more likely to feel self-­directed ageism, whereas those older persons who do not perceive themselves as belonging to the older persons age group are more likely to experience other-directed ageism. Hence, different interventions, which target self-directed ageism versus other-directed ageism might be applicable depending on the level of subjective social group identification.

**Figure 2.**
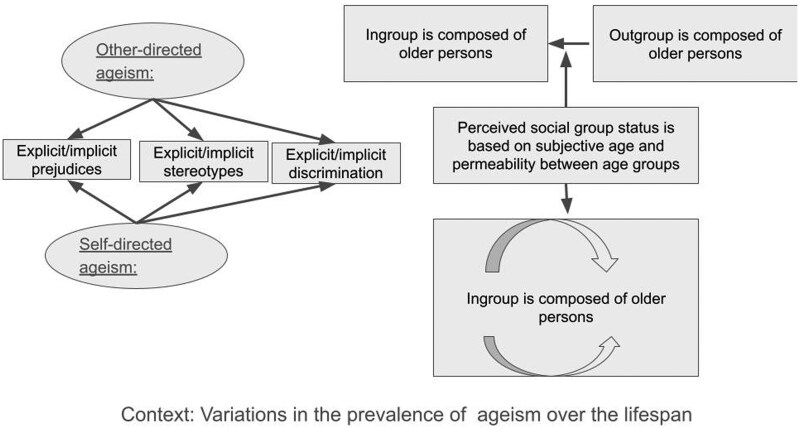
The intragroup approach to addressing ageism.

Figure 2 presents the innovative intragroup approach to understanding ageism in the second half of life. Such an approach moves beyond chronological age to understand ageism. The approach transitions from viewing older persons as merely the targets of ageism, to acknowledging their agency as potential targeters of ageism not only toward themselves, but also toward other older persons. Subjective age and the permeability between age groups are operationalized as capturing one’s subjective social group identification. It is argued here that all three dimensions of ageism, including its implicit component, must be acknowledged and addressed. In addition, the context in which ageism occurs is taken into consideration though no clear hypotheses can be formed at the present time, given the lack of prior research and the Western-centric approach, with most intervention research and theory coming from North America. Due to known variability in the manifestation of ageism in different contexts and cultures, a more nuanced approach that considers the context in which ageism occurs is essential.

#### Alleviating ageism in the second half of life

A personalized intragroup approach to ageism in the second half of life acknowledges the fact that different interventions impact different dimensions of ageism and therefore should be applied to different populations depending on their preintervention levels of ageist stereotypes, prejudices, and discrimination. Interventions can also be differentiated by directly targeting self-directed ageism versus other-directed ageism, with the decision to employ self- or other-directed interventions being guided by one’s subjective age and the permeability between age groups.

Employing a personalized approach, I suggest several potential interventions that address cognitive, behavioral, or emotional dimensions of as well as implicit self-or other-directed ageism. For instance, *cognitive restructuring interventions* are expected to change negative stereotypes of older persons via cognitive mechanisms. Such interventions can either target self-directed or other-directed ageist stereotypes, thus possibly directly impacting self- or other-directed ageism, respectively. The advantage of such techniques concerns the fact that they have been used to address different “isms”(Birtel & Crisp, 2015; Brown et al., 2021), including self-directed ageism (Wolff et al., 2014). Moreover, they have a strong educational component, which has shown to be effective in reducing ageism toward older persons among younger agents of ageism ­(Apriceno & Levy, 2023; Burnes et al., 2019). As to behavioral mechanisms of change: Stemming from the intergroup contact theory (Allport et al., 1954), *intergenerational contact*, under favorable conditions, has strong evidence in reducing ageism toward older persons by younger agents of ageism (Apriceno & Levy, 2023; Burnes et al., 2019). Its effects on self- or ­other-directed ageism among older persons as the agents of ageism have not been studied. However, intergenerational contact had a protective effect in the case of older persons exposed to stereotype threats (Abrams et al., 2006). Intergenerational contact is expected to increase the permeability between groups and, as such, could possibly reduce self-directed ageism. *Intragenerational* (within the group of older persons) *contact*, on the other hand, can result in positive group experiences which reshape one’s views of other older persons as representing “the others,” thus, is possibly more effective in reducing other-­directed ageism. However, this is speculative in the absence of relevant research.

To address self- and other-directed ageism mainly (though not solely) via emotional mechanisms (David & Hayes, 2011), *mindfulness interventions* can be used. Mindfulness interventions do not explicitly differentiate self- versus other-directed ageism but are expected to result in greater acceptance and empathy (Hayes & Wilson, 2003; Lester & Murrell, 2022; Steward, 2022). The theoretical rationale stems from the realization that some losses associated with aging must be accepted, rather than modified (Lev et al., 2018).

As to implicit interventions, there is some evidence to document the benefits of reducing self- and other-directed ageism via *subliminal positive stimuli interventions* depicting older persons in general (Levy et al., 2014) and also alleviating other-­directed ageism in the case of younger persons as the agents of ageism (Ramasamy & Khodabakhsh, 2022). The theoretical rationale of implicit interventions largely stems from the unconscious nature of many ageist stereotypes (Levy & Banaji, 2002) and the need to overcome conscious resistance to change (Dasgupta & Greenwald, 2001). The exact contents of implicit stimuli can either directly address self-ageism via positive subliminal self-directed age stereotypes or other-directed ageism via subliminal positive stereotypes of older persons in general.

### Conclusions and suggestions for future research

The WHO launched a global report on ageism for the first time in history (World Health Organization, 2021). This was in response to a mandate given by 194 countries to act now to alleviate ageism and ensure a society for all ages. As part of the report, two interventions were identified as having enough research evidence: educational interventions concerning older age and aging, highlighting the negative impact of ageism and intergenerational contact between younger and older persons. The combined effect of these two interventions was identified as being particularly effective in alleviating ageist attitudes and prejudices. Although informative, these interventions were tested with younger persons as agents of ageism, and their effectiveness was particularly high among younger age groups (Burnes et al., 2019).

The WHO report completely excluded interventions that address agents of ageism who are in the second half of their life (World Health Organization, 2021). This is even though ageism in the second half of life and especially self-directed ageism received acknowledgement as being highly pervasive and impactful (Kotter-Grühn & Hess, 2012; Levy et al., 2002; ­Tully-Wilson et al., 2021). Interventions to address ageism by older persons in the second half of life directed toward themselves and other older persons were not discussed in the report because of the limited evidence available to support such interventions. Nonetheless, in research with older persons, the importance of addressing self-directed ageism was well-noted. In fact, consistent with already documented research concerning the negative impact of ageism and self-directed ageism among agents of ageism in the second half of their life ­(Kotter-Grühn & Hess, 2012; Levy et al., 2002; Tully-Wilson et al., 2021), older persons argued for the negative impact of self-directed ageism on their own lives (Okun & Ayalon, 2023a).

The WHO concluded its report with an urgent call for researchers to develop interventions to alleviate ageism. Following lessons from personalized medicine (Goetz & Schork, 2018), which relies on the characteristics of the individual patient to match prevention, diagnosis, and treatment decisions, with the understanding that heterogeneity among people must be acknowledged and adequately addressed for optimal outcomes, interventions to alleviate ageism should be highly specific, reflecting a clear understanding concerning what works for whom and why. An intragroup approach, which views ageism as occurring within a single group of older persons, rather than as representing relationships between people of different groups can be a first step toward personalized interventions to alleviate ageism. Although older persons can be classified into a single “ingroup” based on their chronological age, it is essential to recognize their subjective age perceptions and the permeability of their social relations with different age groups to identify adequate interventions to alleviate intragroup stereotypes, prejudices, and discrimination against other older persons (e.g., other-directed ageism) and themselves (e.g., self-directed ageism).

The proposed theoretical framework draws on and expands existing theories, such as stereotype embodiment theory (Levy, 2009) and social identity theory (Tajfel, 1974; Tajfel & Turner, 2004). The intragroup approach adds to the current body of knowledge by stressing the role of older persons as potential agents of ageism, rather than as merely victims or targets of ageism. An intragroup approach makes a clear distinction between ingroup and outgroup members based on chronological age and acknowledges the role of subjective social group identification in self- and other-directed ageism. The approach also considers the multidimensional nature of ageism, including its implicit component. Although no clear predictions are made regarding the context in which interventions take place, the potential importance of context is acknowledged. Likewise, although the proposed theoretical framework does not make clear predictions concerning intersectionality (e.g., the holding of multiple social attributes), its focus on preintervention characteristics of the agent of ageism to match interventions to participants is a step forward toward acknowledging heterogeneity in intervention selection. Intersectionality may also be acknowledged in relation to the target of ageism, as clearly ageism is experienced differently in intersection with other attributes, such as sex or ethnicity/race. Hence, it is possible that knowledge from research addressing other types of “ism” such as sexism or racism can guide future interventions to address ageism toward older persons.

A central idea of the intragroup approach concerns the fact that less clear boundaries between age groups can result in reduced ageism. However, this remains a theoretical hypothesis that needs to be examined empirically. Likewise, the role of context in determining the type and effectiveness of ageism interventions is still under-established empirically. Nonetheless, the proposed intragroup approach is expected to result in more effective interventions to alleviate self- and other-directed ageism as well as in a more nuanced understanding and predictions of change in ageism in the second half of life. Future research will benefit from empirically testing the predictions made by the intragroup approach to transition to a society for all ages.

## Data Availability

This article does not report data and therefore the preregistration and data availability requirements are not applicable.
